# Supplementary material for: FastqPuri: high-performance preprocessing of RNA-seq data
Source: BMC Bioinformatics. 2019 May 3;20:226. doi: 10.1186/s12859-019-2799-0 (PMC6500068; doi:10.1186/s12859-019-2799-0)
Supplement: Supplementary file 2 — Archive of FastqPuri. Archive containing all files needed to install and run FastqPuri v1.0.6. Date stamp March 22, 2019. (GZ 47,819 kb) [file 12859_2019_2799_MOESM2_ESM.gz › FastqPuri-1.0.6/html/struct__iparam__Qreport.html]

FastqPuri: \_iparam\_Qreport Struct Reference


FastqPuri

Public Attributes |
List of all members

\_iparam\_Qreport Struct Reference

contains Qreport input parameters
More...

`#include <init_Qreport.h>`

|  |  |
| --- | --- |
| Public Attributes | |
| char \* | inputfile |
|  | |
| char | outputfilebin [MAX\_FILENAME] |
|  | |
| char | outputfilehtml [MAX\_FILENAME] |
|  | |
| char | outputfileinfo [MAX\_FILENAME] |
|  | |
| int | nQ |
|  | |
| int | ntiles |
|  | |
| int | minQ |
|  | |
| int | read\_len |
|  | |
| int | filter |
|  | |
| int | one\_read\_len |
|  | |

## Detailed Description

contains Qreport input parameters

## Member Data Documentation

## ◆ filter

int \_iparam\_Qreport::filter

0 original data, 1 this tool filtered data, 2 other tool filtered data

## ◆ inputfile

char\* \_iparam\_Qreport::inputfile

Inputfile name

## ◆ minQ

int \_iparam\_Qreport::minQ

minimum Quality allowed 0 - 45

## ◆ nQ

int \_iparam\_Qreport::nQ

# different quality values (default is 46)

## ◆ ntiles

int \_iparam\_Qreport::ntiles

# tiles (default is 96)

## ◆ one\_read\_len

int \_iparam\_Qreport::one\_read\_len

1 all reads of equal length 0 reads have different lengths.

## ◆ outputfilebin

char \_iparam\_Qreport::outputfilebin[MAX\_FILENAME]

Binary outputfile name.

## ◆ outputfilehtml

char \_iparam\_Qreport::outputfilehtml[MAX\_FILENAME]

html outputfile name

## ◆ outputfileinfo

char \_iparam\_Qreport::outputfileinfo[MAX\_FILENAME]

Info outputfile name

## ◆ read\_len

int \_iparam\_Qreport::read\_len

original read length

---

The documentation for this struct was generated from the following file:

- include/init\_Qreport.h


---

Generated on Mon Mar 19 2018 23:42:01 for FastqPuri by  

 1.8.14
